# Liver fat accumulation in response to overfeeding with a high-fat diet: a comparison between South Asian and Caucasian men

**DOI:** 10.1186/s12986-015-0015-4

**Published:** 2015-05-20

**Authors:** Siti N. Wulan, Vera B. Schrauwen-Hinderling, Klaas R. Westerterp, Guy Plasqui

**Affiliations:** Department of Human Biology, Nutrition and Toxicology Research Institute (NUTRIM) - School for Nutrition Toxicology and Metabolism, Maastricht University Medical Center (MUMC+), Universiteitssingel 50, PO.Box 616, 6200MD Maastricht, The Netherlands; Laboratory of Food Quality and Nutrition, Department of Food and Agricultural Product Technology, Faculty of Agricultural Technology, Brawijaya University, Malang, East Java Indonesia; Department of Radiology, Maastricht Academic Hospital, Maastricht University Medical Center (MUMC+), Maastricht, The Netherlands

**Keywords:** Liver fat content, Body fat distribution, Overfeeding, South Asian men

## Abstract

**Background:**

South Asians were reported to have a higher liver fat content as compared to BMI-matched Caucasians. This study compared the increase in liver fat content in response to overfeeding with a high fat diet in South Asian and Caucasian men when matched for body fat percentage.

**Methods:**

Ten South Asian men (BMI 18–29 kg/m^2^) and 10 Caucasian men (BMI 22–33 kg/m^2^), aged 20–40 y, matched for body fat percentage, were included. A weight maintenance diet was given for 3 days based on the individual energy requirement. Individual energy requirement of the subjects was calculated based on their body composition (measured by hydro densitometry and deuterium dilution) and activity counts (accelerometer). Liver fat content was measured before and after 4 days of overfeeding (50 % excess energy need) with a high fat diet (60 % energy from fat). Fat distribution was measured by anthropometry and an MRI scan of the abdomen while liver fat content using 1H-MRS.

**Results:**

While having a similar body fat % (P = 0.58), South Asians had a lower BMI (P = 0.04) than Caucasians. Liver fat content at baseline did not differ between ethnicities (P = 0.48) and was associated with visceral fat area (P = 0.002, R^2^ = 0.56) but not with ethnicity (P = 0.13). Overfeeding with a high fat diet significantly increased liver fat (P = 0.01) but the increase did not differ between ethnicities (P = 0.47). There was no difference in the total abdominal fat area (P = 0.37), subcutaneous abdominal fat area (P = 0.18) and visceral fat area (VAT, P = 0.32). However as a percentage of the total abdominal fat area, VAT was higher in South Asians (P = 0.003).

**Conclusion:**

Despite a relatively higher percentage of visceral fat area, liver fat increased similarly in South Asian and Caucasian men in response to overfeeding with a high fat diet.

**Trial registration:**

The study was registered in the public trial registry www.ccmo.nl No. NL31217.068.10.

## Background

The epidemic of overweight and obesity is one of the major public health problems not only in western countries but also in a number of Asian countries [1, 2]. Globally, adult obesity is more common than under-nutrition affecting around 475 million adults who are obese, with over twice that number overweight. When Asian-specific cut-off points for the definition of obesity (body mass index >28 kg/m^2^) are taken into account, the number of adults considered obese globally is over 600 million [3].

Differences in body composition between Asians and Caucasians matched for sex, BMI and age have been reported in several comparative studies. We [4] and others [5–9] found that Asians have a higher body fat percentage and a lower fat-free mass/appendicular skeletal muscle mass [10–12] compared to that of Caucasians. In addition, when the fat-free mass was corrected for body height, Asians were shown to have the lowest fat-free mass index (FFMI, in kg/m^2^) of ethnicities such as Hispanics, African-Americans and Caucasians [13].

Among Asians, South Asians (people from Indian sub-continent) have the most pronounced difference in body fat [14] and an increased ectopic fat deposition in the liver [15] and muscle [5] compared to those in BMI-matched European Caucasians. It is hypothesized that South Asians have a lower capacity to store fat in subcutaneous adipocytes than Caucasians [16]. Excess fat may therefore overflow to the ectopic compartments, such as in the liver [15]. Thus, South Asians may be more susceptible to the negative effects of a high-fat diet. Ectopic fat accumulation in the liver has been shown to be associated with insulin resistance in Caucasians [17, 18] and South Asians [15].

It is well known that genetics may play a role and the interaction with environmental factors such as changes in lifestyle could increase the risk of developing the metabolic syndrome [19]. In Asia, the consumption of fat and added sugar in the diet have increased in recent decades [20]. The effect of a high fat diet on ectopic fat accumulation in the liver in Caucasian populations has been documented in some studies [21–23], whereas IMCL (intramyocellular lipids) content increased in some studies [24] but not in others [21]. Van Herpen et al. [21] conducted a 3-week period of high fat isocaloric diet study with 60 % energy from fat and reported that the increase in liver fat was observed after 1 week with no further increase in the following weeks. In the present study, we induced liver fat accumulation with a high fat diet (60 % energy from fat) in a shorter time by overfeeding with 50 % excess energy than the requirement. Whether a high fat diet affects liver fat accumulation in South Asians and Caucasian similarly is unknown. A previous cross-sectional study [15] reported a higher liver fat content in South Asians compared to Caucasians after correcting for BMI, sex and age. As there is an ethnic-specific BMI-body fat percentage relationship [8], in the present study South Asian and Caucasian men were matched for body fat percentage instead of BMI.

The objectives of the present study were to compare liver fat content at baseline between South Asian and Caucasian men matched for body fat percentage and to compare the increase in fat accumulation in the liver in response to short-term overfeeding with a high fat diet.

## Methods

### Study subjects

Subjects were 10 healthy adult non-diabetic South Asian men and 10 Caucasian men, matched for body fat percentage. The number of subjects was determined based on previous studies showing an increase in liver fat content after short-term high fat feeding in Caucasian subjects [21, 23]. Subject characteristics are presented in Table 1. Asian subjects had 4 grandparents from South Asia, while Caucasian subjects were non-Hispanic Europeans. South Asian subjects were students who temporarily living in Europe, and were not recruited from the European population. Subjects were selected based on the following inclusion criteria: healthy, not having metabolic diseases (diabetes or cardiovascular diseases), not using medication, aged between 20 and 40 years old with a body mass index between 18–29 kg/m^2^ for South Asians and 22–33 kg/m^2^ for Caucasians, having a stable body weight for the last three months, having no-low-or moderate alcohol intake, not being on a diet and no athletes. All subjects received verbal and written information before giving their consent to participate in the study. The study was approved by Medical Ethics Committee of Maastricht University Medical Centre, MEC No. 10-3-013 and registered in the public trial registry (www.ccmo.nl No. NL31217.068.10).

**Table 1:** Subjects’ characteristics^a^

Characteristics	South Asian	Caucasian	P
N	10	10	-
Age^b^ (y)	27 ± 2	24 ± 2	0.001
Body weight (kg)	68.9 ± 7.4	88.3 ± 18.1	0.009
BMI (kg/m^2^)	23.3 ± 3.0	27.0 ± 4.2	0.04
Fat mass (%)	25.5 ± 6.4	23.7 ± 7.5	0.58
Fat-free mass (kg)	51.0 ± 3.7	66.5 ± 8.5	0.001
Waist circumference (cm)	85.9 ± 7.7	96.0 ± 12.0	0.05
Hip circumference (cm)	97.0 ± 5.5	105.4 ± 8.7	0.03
Waist to hip ratio	0.9 ± 0.04	0.91 ± 0.06	0.32
VO_2_ max corrected for FFM^c^ (ml O_2_/min)	2780 ± 265	3249 ± 152	0.15
Physical activity accelerometer^b^ (10^3^counts/d)	1123 ± 239	1304 ± 442	0.68

^a^Differences between groups were analyzed using Independent samples *t*-test when data in both groups were normally distributed according to normality test Kolmogorov-Smirnov and Shapiro-Wilk

^b^For age and physical activity count, data of the Caucasian group were not normally distributed, differences between groups were assessed using non-parametric test Mann–Whitney *U* test

^c^VO_2_ max corrected for FFM, was analyzed using ANCOVA with FFM as a co variable

### Study design

This study was a diet-intervention study in free-living conditions. Body composition was measured at baseline to have a matched body fat percentage between two ethnic groups. Cardio-respiratory fitness was measured prior to the diet intervention. Daily physical activity was measured for 7 consecutive days with an accelerometer. A diet to maintain energy balance for 3 days was prepared on the basis of fat-free mass and the daily physical activity counts of each subject. Baseline measurement of hepatic fat content was performed afterwards. The overfeeding with a high fat diet for an interval of 4 days was started right after the baseline measurement and hepatic fat content was measured again after the overfeeding interval. All measurements were carried out at the Metabolic Research Unit Maastricht (MRUM), Maastricht University, Maastricht, The Netherlands.

### Body composition

Body composition was determined according to a 3-compartment model based on body weight, body volume and total body water. Body weight and body volume were determined in the morning, in the fasting state. Body volume was determined by hydro-densitometry with simultaneous measurement of residual lung volume using the helium dilution technique. Total body water was determined with deuterium dilution according to the Maastricht protocol [25]. Body composition was calculated from body density and total body water using the equation of Siri [26].

### Anthropometry

Anthropometric measures were performed at the same time as body composition. Waist circumference was measured using a circumference measuring tape (Seca 201, United Kingdom) at the umbilical, while subject standing in very light clothing. Hip circumference was measured as the largest circumference between waist and thighs [27]. Skinfold thickness of the biceps, triceps, subscapular and suprailiac was determined using Harpenden skinfold caliper (Body Care, England). Anthropometry measures were performed using The NHANES body measurement guidelines [28].

### Cardio-respiratory fitness

Physical fitness was assessed with an incremental test on a bicycle ergometer using the protocol of Kuipers et al. [29]. During the test, O_2_-consumption and CO_2_-production were measured continuously and heart rate was monitored (Polar heart rate monitor, Polar Electro Oy, Kempele, Finland). After a warming up of 5 min at 100 Watt (W) for men, workload was increased with 50 W every 2.5 min. When heart rate (HR) reached a value of 35 beats per min (bpm) below the age predicted maximal HR (220 bpm - age) or the respiratory quotient (RQ = CO_2_-production/O_2_-consumption) exceeded 1, workload was increased with 25 W every 2.5 min until exhaustion. VO_2_ max was determined by averaging the last few points of maximum O_2_ consumption. Cardio-respiratory fitness was defined as VO_2_ max divided by FFM.

### Daily physical activity level

The daily physical activity level (PAL) was measured using a Direct Life triaxial accelerometer for movement registration (Tracmor_D_) (Philips New Wellness Solutions; http://www.directlife.philips.com). The device is a small (3.2 × 3.2 × 0.5 cm), light-weight (12.5 g) instrument. The accelerometer was attached to the lower back by means of an elastic belt. It registered accelerations minute by minute, in the mediolateral (x-axis), longitudinal (y-axis) and anterioposterior (z-axis) of the trunk as described elsewhere [30]. Subjects were instructed to wear the accelerometer for 7 consecutive days, during waking hours except during water activities. Subjects were advised to maintain their habitual physical activity level and not to perform any strenuous physical activity during the diet intervention. Tracmor_D_ output was expressed as activity counts/min. The Tracmor_D_ activity counts/min were summed over the entire monitoring period and divided by the number of monitoring days to determine the average Tracmor_D_ counts per day (counts/d). Daily PAL was calculated based on the activity counts/d with the formula, PAL = 1.354 + 256 × 10^−9^× counts/d [30]. Daily total energy expenditure was calculated with the formula of Bonomi et al., TEE accelerometer = 0.04 + 0.17 FFM + 1.67 × 10^−6^ × counts/d [30] by including activity counts/d (from the accelerometer) and fat-free mass (FFM, from the body composition measurement).

### Energy intake

The weight maintenance diet to be consumed at home for 3 days before the baseline measurement was calculated on the basis of TEE. TEE was calculated by the formula of Bonomi et al. [30] as described above. The macronutrients distribution of the diet prior to baseline measurement was 30 % fat, 55 % carbohydrate and 15 % protein.

The overfeeding with a high fat diet was prepared with 50 % excess energy above the requirement [31]. The macronutrients composition of the high fat diet was 60 % fat, 25 % carbohydrate and 15 % protein [24, 32]. Fatty acids composition of the diet was 40 % saturated fatty acids and 60 % unsaturated fatty acids.

A written instruction was given to prepare the diet at home. During the weight maintenance, subjects were provided with the diet in an excess amount than TEE and were allowed to eat more or less from the diet prescribed, according to what they needed (*ad libitum*). Any additional intake from those prescribed foods was recorded. All unfinished foods were collected and returned to the university, to calculate actual energy intake. During the overfeeding period, subjects were asked to finish all the foods prescribed, but otherwise noted down and returned the unfinished foods. The diet consisted of normal ready-to-eat foods combining a typical Western and Asian diet. Foods were selected by reviewing the ingredients content to ensure there was no/limited effect of certain ingredients on fat oxidation (such as spices). During the high fat diet period, subjects were also provided with decaffeinated coffee and fruit tea, as caffeine was also reported to increase fat oxidation. Alcohol intake was limited during the diet intervention (only one serving per day if needed) and was not allowed within 2 days prior to the liver fat measurement.

### Abdominal fat measurement

Fat in the abdominal compartment was measured using a 3.0 Tesla MRI scanner (Achieva, Philips Healthcare, Best, The Netherlands) with a body coil. A single MRI slice on the umbilical level [33] was acquired using an axial T1 weighted spin-echo sequence in breath hold with following parameters: echo time of 15 ms, repetition time of 500 ms, matrix size of 180 × 96 and slice thickness of 8 mm. Analysis of the abdominal MR images was performed using OsiriX DICOM viewer software. A threshold value was chosen for separation of lean and adipose tissue. Manual segmentation by the region-growing tool was used to segment the area of total abdominal fat (TAT) and region of interest (ROI) borderline for subcutaneous abdominal fat (SAT), whereas visceral fat (VAT) was calculated by subtracting SAT from TAT.

### Hepatic lipid content

Lipid accumulation in the liver was measured before and after overfeeding with a high fat diet. All measurements were performed on a 3.0 T Philips Achieva scanner (Philips Healthcare, Best, The Netherlands) using a SENSE-cardiac coil [34]. A single voxel of 20 × 20 × 20 mm^3^ was positioned in the right liver lobe, avoiding large biliary or vascular structures [34]. Spectra were acquired using a point-resolved spectroscopy sequence PRESS [34] with repetition time of 4000 ms, echo time of 33 ms, and number of averages of 64. To minimize the motion artifacts, subjects were asked to breathe in the rhythm of the measurement and to be at end-expiration during acquisition of spectra [35]. To determine the intensity of the lipid peak, the water signal was suppressed using frequency-selective prepulses. The unsuppressed water resonance was used as internal reference (number of averages = 16). The spectra were fitted with AMARES [36] in the jMRUI software [37]. Values are given as T2-corrected ratios of the CH_2_ peak, relative to the unsuppressed water resonance (as percentage) according to Hamilton et al. [38].

### Statistical analysis

Data are presented as means and SDs. Data were first tested for normal distribution by using normality test Kolmogorov-Smirnov and Shapiro-Wilk. When normality was met, statistical comparison of the subjects’ baseline characteristics and body fat distribution between ethnic groups was performed using independent sample *t*-test. Non-normally distributed data were compared using the non-parametric Mann-Whitney *U* test. ANOVA repeated measures were performed to compare differences in changes in liver fat accumulation before and after overfeeding with a high fat diet within and between groups. ANCOVA analysis and multiple regression analysis were applied to assess the main effect of ethnicity on parameters of interests by including potential covariates (determinants). The SPSS program version 20 (SPSS, Chicago, IL) was used for statistical analysis, and statistical significance was set as p < 0.05.

## Results

### Subject characteristics

South Asian subjects were Indian (n = 8) and Pakistani (n = 2). Caucasian subjects were Dutch (n = 3), Germans (n = 2), French (n = 1), British (n = 1), Danish (n = 1), Polish (n = 1) and Icelander (n = 1). South Asians were measured within 3 y of their stay in The Netherlands (n = 5) and within 1 y (n = 5). Subjects’ characteristics are presented in Table 1. South Asian subjects were slightly older than Caucasians and had a significantly lower BMI (*P = 0.04*), but there was no difference in body fat percentage (*P = 0.58*). As consistently reported by many studies, South Asian men had a lower fat-free mass *(P = 0.001)* compared to Caucasian men, and a lower fat-free mass per square meter of height (17 kg/m^2^ versus 20 kg/m^2^).

Waist and hip circumference were higher in Caucasians than Asians, resulting in no difference in waist to hip ratio between ethnicities (*P = 0.32*). There was no difference in cardio-respiratory fitness corrected for fat-free mass between ethnicities (*P = 0.15*) and nor was physical activity (*P = 0.68*).

### Body fat distribution

Body fat distribution of South Asian and Caucasian men are presented in Table 2. Caucasian men had a slightly higher absolute fat mass and fat mass per square meter of height but this was not significantly different (*P = 0.85* and *P = 0.65* respectively). Body composition of our two populations was further characterized, by calculating the fat mass to fat-free mass ratio [39]. In our study, the matching procedure in body fat percentage resulted in no difference in the fat mass to fat-free mass ratio *(P = 0.63).*

**Table 2:** Body fat distribution^a, b^

Characteristics	South Asian	Caucasian	P
N	10	10	-
Fat mass (kg)	17.9 ± 6.4	22.0 ± 11.5	0.85
Fat mass to fat-free mass ratio	0.35 ± 0.11	0.32 ± 0.14	0.63
Fat mass index (kg/m^2^)	6.1 ± 2.1	6.6 ± 3.1	0.65
Total abdominal fat area, TAT^c^ (cm^2^)	241.9 ± 90.7	296.9 ± 166.0	0.37
Subcutaneous abdominal fat area, SAT (cm^2^)	164.2 ± 65.6	230.4 ± 132.9	0.18
Subcutaneous fat (% TAT)	67.3 ± 3.6	76.7 ± 7.5	0.003
Visceral fat area, VAT (cm^2^)	77.8 ± 26.1	66.5 ± 42.5	0.32
Visceral fat (% TAT)	32.7 ± 3.6	23.3 ± 7.5	0.003
VAT/SAT ratio	0.49 ± 0.1	0.32 ± 0.1	0.003
Biceps skinfold (mm)	3.9 ± 2.0	3.2 ± 0.6	0.91
Triceps skinfold (mm)	7.7 ± 3.1	7.2 ± 3.9	0.19
Subscapular skinfold (mm)	19.2 ± 8.8	16.0 ± 6.5	0.36
Suprailiac skinfold (mm)	13.3 ± 6.6	12.2 ± 5.4	0.69

^a^Differences between groups were analyzed using Independent samples *t*-test when data in both groups were normally distributed according to normality test Kolmogorov-Smirnov and Shapiro-Wilk

^b^Fat mass (kg), visceral fat area, biceps and triceps skin fold were not normally distributed in one or both groups, differences between groups were assessed using non-parametric test Mann–Whitney *U* test

^c^Abdominal fat scan was performed at the umbilical except in one subject of the South Asian group, in this subject abdominal fat scan was performed at the sagittal lumbar spine L_3_/L_4_ instead of the umbilical. Excluding this subject did not change the resulting P values for TAT, SAT, VAT, percentage VAT and VAT/SAT ratio (0.36; 0.18; 0.36; 0.004 and 0.004 respectively)

Abdominal obesity as shown by total abdominal fat area (TAT) and subcutaneous abdominal fat area (SAT) was slightly higher in Caucasians, whereas visceral fat area (VAT) was slightly higher in South Asians but did not reach statistical significance (*P = 0.37, P =0.18 and P = 0.32* respectively). This may be due to the larger variation in the Caucasian group. However, as a percentage of the total abdominal fat area, VAT was higher in South Asians (*P = 0.003*) and so was the VAT to SAT ratio (*P = 0.003*). The abdominal fat scan was performed at the umbilical, except in one subject of the South Asian group, in this subject abdominal fat scan was performed at the sagital lumbar spine L_3_/L_4_ instead of the umbilical. Excluding this subject did not change the resulting P values for TAT, SAT, VAT, percentage VAT and VAT/SAT ratio (*0.36; 0.18; 0.36; 0.004* and *0.004* respectively).

Fat in the extremities measured as biceps and triceps skinfold did not differ between ethnicities (*P = 0.91* and *P = 0.19* respectively). Truncal fat measured as subscapular skinfold was not different between groups (P = 0.36) nor was the suprailliac skinfold (*P = 0.69*).

### Diet composition and compliance

There was no difference in compliance to the diet. Energy intake, as percentage overfeeding achieved, was 43 % and 52 % exceeding the requirement (*P = 0.16*) for South Asians and Caucasians respectively. Macronutrients composition of the actual energy intake during overfeeding was similar; with carbohydrate (26.2 % versus 26.4 %, *P = 0.85*), protein (14.6 % versus 14.9 %, *P = 0.20*) and fat (59.3 % versus 58.8 %, *P = 0.53*) for South Asians and Caucasians respectively. There was no difference in the proportion of saturated and unsaturated fatty acids in the diet (*P = 0.49*).

### Hepatic fat content

**Fig. 1:**
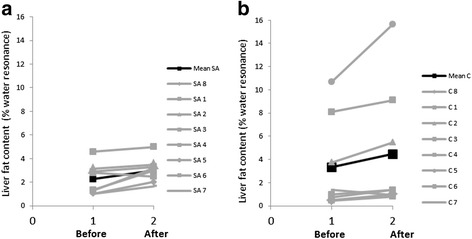
Individual (grey line) and the mean (black line) response of liver fat content to overfeeding with a high fat diet in South Asians (**a**) and Caucasians (**b**). Liver fat content before and after overfeeding with a high fat diet were assessed using ANOVA repeated measure. Data were available from 8 South Asians and 8 Caucasians matched for body fat percentage (25.0 ± 5.4 % and 23.2 ± 6.3 % for South Asian and Caucasian respectively, P = 0.53). Overfeeding with a high fat diet increased liver fat content (P = 0.01) but the increase did not differ between ethnicities (P = 0.47). SA: South Asian, C: Caucasian

Liver fat content before and after short-term overfeeding with a high fat diet is shown in Fig. 1. Data were available for 8 South Asians and 8 Caucasians matched for body fat percentage (*P = 0.53*). In the South Asian group, one had to be excluded because of poor signal to noise ratio in the spectra. In the Caucasian group, one subject could not undergo the measurement due to a technical problem. Therefore, these subjects were excluded from the analysis along with their body fat matched counterparts in the other group respectively.

**Fig. 2:**
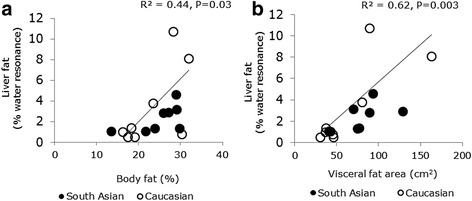
The association between liver fat at baseline with body fat percentage (**a**) and the association between liver fat at baseline with visceral fat area (**b**). Liver fat at baseline was associated with body fat percentage (R^2^ = 0.44, P = 0.03). Liver fat at baseline had a stronger association with visceral fat area (R^2^ = 0. 62, P = 0.003). In a multiple regression analysis, visceral fat area was the significant predictor of baseline liver fat (R^2^ = 0.56, P = 0.002) and no effect of ethncity was found (P = 0.13). Excluding the outlier, did not change the significant association between body fat percentage and baseline liver fat content in Fig. 2a (R^2^ = 0.36, P = 0.018) and the significant association between visceral fat area and baseline liver fat content in Fig. 2b (R^2^ = 0.76, P = 0.001)

Liver fat content at baseline did not differ between ethnicities (*P = 0.48*). Overfeeding with a high fat diet significantly increased liver fat content (*P = 0.01*) but the increase did not differ between ethnicities (*P = 0.47*). The mean increase was 33 % and 34 % for South Asians and Caucasians respectively. We assessed the association between liver fat at baseline and body fat percentage, it turned out that liver fat at baseline was positively associated with body fat percentage (*R*^*2*^ *= 0.44, P = 0.03*, Fig. 2a). Furthermore, we assessed the effect of ethnicity on the baseline liver fat content with body fat percentage as a covariate (ANCOVA analysis) and found that baseline liver fat content was associated with body fat percentage (*P = 0.02*) but not with ethnicity (*P = 0.21*). We assessed the association between liver fat at baseline with visceral fat area, and found that liver fat at baseline had a stronger association with visceral fat area (*R*^*2*^ *= 0.62, P = 0.003*, Fig. 2b). In a multiple regression analysis by including ethnicity in the model, visceral fat area was found to be the significant predictor of liver fat content at baseline (*P = 0.002, R*^*2*^ *= 0.56*) and not ethnicity (*P = 0.13*). There was an outlier in Fig. 2a and b. Excluding the outlier, did not change the significant association between body fat percentage and baseline liver fat content in Fig. 2a (*R*^*2*^ *= 0.36, P = 0.018*) and the significant association between visceral fat area and baseline liver fat content in Fig. 2b (*R*^*2*^ *= 0.76, P = 0.001*).

## Discussion

We found that South Asian and Caucasian men with the same body fat percentage (not the same BMI) showed no difference in the baseline liver fat content. Furthermore, visceral fat area was found to be the significant predictor of liver fat content at baseline. Liver fat content increased similarly in both groups in response to short term overfeeding with a high fat diet.

Earlier cross-sectional studies reported higher liver fat content in South Asians when compared to BMI-matched Caucasians [15], suggesting a higher susceptibility to ectopic fat storage in Asians. It is known that, due to differences in body composition, matching for BMI will result in a higher body fat percentage in the Asian group, which may be a confounding factor. Therefore, in the current study, we chose to match subjects with respect to whole body fat percentage, as determined by a three-compartment model. When these groups, with similar body fat percentage, were compared, hepatic lipid content was similar, and in both groups, body fat percentage and the visceral adipose tissue area (which was also similar between groups) were predictive for baseline liver fat content. This is in line with earlier studies, where visceral fat has been reported to be associated with liver fat content in Caucasian populations [18, 40, 41] as well as a South Asian population [15].

It was also reported that South Asians might be more susceptible to the negative metabolic effects of high-fat high-caloric diets. Also here, the difference in body fat percentage between Asians and Caucasians (when matched for BMI) may have played a role. To investigate whether Asians still have higher susceptibility to a high energy, high-fat diet, when controlled for body fat percentage, we investigated hepatic fat storage after overfeeding with a high fat diet for 4 days in groups matched for body fat percentage. We expected to create a massive positive energy balance that favors fat accumulation rather than fat oxidation.

Earlier studies already showed that high fat diets can increase liver fat content in a relatively short period of time. A dietary intervention with an isocaloric high fat diet [21, 23] resulted in an increase in liver fat content by 35 % after 2 weeks in obese Caucasian women [23] and by 17 % after 3 weeks in overweight Caucasian men [21]. In the latter study, fat accumulation was observed after one week with no further increase in the following weeks, suggesting an adaptation [21]. This is also in accordance with a study in healthy male Caucasians by van der Meer et al. [22] showing that a 2–3 fold increase in liver fat content had already occurred after 3 days consumption of supplemental cream (800 ml, 280 g fat) added to the regular diet.

Here, we conducted a short-term intervention with a high fat diet (4 days), by overfeeding subjects with 50 % energy above the individual requirements and 60 % of energy from fat. The mean increase in liver fat content was 33 and 34 % in South Asians and Caucasians respectively. Thus in South Asian and Caucasian men with the same body fat percentage, and similar liver fat content at baseline, the increase in liver fat in response to short-term overfeeding with a high fat diet was similar. The inter-individual variability in the baseline liver fat is rather high, especially in the Caucasian group and is partially explained by the range of body fat percentages included (range from 17 to 31 %, see also Fig. 1). The increase in liver fat content is modest, but very consistent and highly significant. The inter-individual variability in the percentage changes in liver fat content is higher than differences between ethnicities. Therefore, when carefully matched for body fat percentage (rather than BMI) we find no indication of Asian subjects being more susceptible to overfeeding.

In a study with very similar set-up, we found insulin levels to be increased in both, Asians and Caucasians [42], which may be favoring hepatic fat storage and therefore underlie the present findings. During overfeeding, the postprandial state, including high insulin levels, is slightly extended as a result of extended meal consumption [42]. Insulin was also reported to suppress hepatic lipid oxidation [43] and to promote the synthesis and storage of triglycerides in the liver [44].

Interestingly, even though absolute amount of visceral adipose tissue was similar between the two groups, it is striking that due to smaller body size in Asians, the visceral adipose tissue depot represents a higher percentage of total abdominal adipose tissue. It was hypothesized that the high prevalence of metabolic diseases in South Asians might be attributed to a smaller subcutaneous adipose tissue compartment and a relatively enlarged VAT. As obesity develops, South Asians could exceed the storage capacity of subcutaneous adipose tissue before Caucasians do and develop metabolic complications [16]. Our data confirm that in the abdominal region, South Asians have a relatively enlarged VAT and a relatively smaller subcutaneous adipose tissue compartment. Lear et al. [45] reported that throughout a range of total body fat mass, South Asians had less VAT with total body fat > 37.4 kg, but more VAT when total body fat was below 37.4 kg than did Europeans, after adjusted for age, sex and household income. In our study, South Asian men had a range of absolute fat mass between 7–29 kg and thus the relatively higher VAT was consistent with a previous study [45]. However, our data showed that despite a relatively higher percentage of VAT area in South Asians, liver fat content at baseline did not differ between ethnicities and also the response to overfeeding was very similar in the two groups.

The limitation of our study is that we induced exposure to excess FFA from the diet in a short-term period, thus it cannot elucidate the differences, which may be observed in a longer period. Additionally, the low number of subjects may not represent the general population in South Asian and Caucasian and only young men were investigated. Although we matched the subjects for body fat percentage, we found variation in the baseline liver fat content and the variation was larger in Caucasians which was not ideal. Taken into account the limitations, this was a well-controlled dietary intervention study using state of the art techniques to investigate liver fat content and body composition in response to overfeeding and no such studies have been performed comparing two ethnicities.

## Conclusions

In summary, when groups are matched for body fat percentage, no differences in liver fat were found at baseline between South Asians and Caucasians and both groups showed a similar increase in hepatic lipid content in response to a hypercaloric, high-fat diet. This suggests that differences between ethnicities that were reported so far may (at least partially) be ascribed to differences in body fat percentage.

Future studies to compare the response of different ethnicities to dietary intervention may be approached by matching body fat percentage within a closer range, by including only lean or only overweight subjects, to avoid large inter-individual variation.

## Abbreviations

BMI: Body mass index
FFA: Free-fatty acids
FFM: Fat-free mass
FM: Fat mass
IMCL: Intramyocellular lipid
1H-MRS: Proton-magnetic resonance spectroscopy
MRI: Magnetic resonance imaging
PAL: Physical activity level
SAT: Subcutaneous abdominal adipose tissue
TAT: Total abdominal adipose tissue
TEE: Total energy expenditure
VAT: Visceral adipose tissue

## References

[1] Popkin BM (2006). Global nutrition dynamics: the world is shifting rapidly toward a diet linked with noncommunicable diseases. Am J Clin Nutr.

[2] Subramanian SV, Perkins JM, Ozaltin E, Davey Smith G. Weight of nations: a socioeconomic analysis of women in low-to middle-income countries. Am J Clin Nutr. 2011;93:413–21. 10.3945/ajcn.110.004820 PMC3021433 21068343

[3] Kelly T, Yang W, Chen CS, Reynolds K, He J (2008). Global burden of obesity in 2005 and projections to 2030. Int J Obes (Lond).

[4] Wulan SN, Westerterp KR, Plasqui G. Dietary and 24-h fat oxidation in Asians and whites who differ in body composition. Am J Clin Nutr. 2012;95:1335–41. 10.3945/ajcn.111.031369 22552026

[5] Forouhi NG, Jenkinson G, Thomas EL, Mullick S, Mierisova S, Bhonsle U (1999). Relation of triglyceride stores in skeletal muscle cells to central obesity and insulin sensitivity in European and South Asian men. Diabetologia.

[6] Deurenberg P, Deurenberg-Yap M (2001). Differences in body-composition assumptions across ethnic groups: practical consequences. Curr Opin Clin Nutr Metab Care.

[7] Deurenberg P, Deurenberg-Yap M (2003). Validity of body composition methods across ethnic population groups. Forum Nutr.

[8] Deurenberg P, Deurenberg-Yap M, Guricci S (2002). Asians are different from Caucasians and from each other in their body mass index/body fat per cent relationship. Obes Rev.

[9] Chang CJ, Wu CH, Chang CS, Yao WJ, Yang YC, Wu JS (2003). Low body mass index but high percent body fat in Taiwanese subjects: implications of obesity cutoffs. Int J Obes Relat Metab Disord.

[10] Rush EC, Goedecke JH, Jennings C, Micklesfield L, Dugas L, Lambert EV (2007). BMI, fat and muscle differences in urban women of five ethnicities from two countries. Int J Obes (Lond).

[11] Rush EC, Freitas I, Plank LD (2009). Body size, body composition and fat distribution: comparative analysis of European, Maori, Pacific Island and Asian Indian adults. Br J Nutr.

[12] Sampei MA, Novo NF, Juliano Y, Sigulem DM (2008). Anthropometry and body composition in ethnic Japanese and Caucasian adolescent boys. Pediatr Int.

[13] Hull HR, Thornton J, Wang J, Pierson RN, Jr., Kaleem Z, Pi-Sunyer X, et al. Fat-free mass index: changes and race/ethnic differences in adulthood. Int J Obes (Lond). 2010;35:121–7. 10.1038/ijo.2010.111 PMC3306818 20531353

[14] Deurenberg-Yap M, Schmidt G, van Staveren WA, Hautvast JG, Deurenberg P (2001). Body fat measurement among Singaporean Chinese, Malays and Indians: a comparative study using a four-compartment model and different two-compartment models. Br J Nutr.

[15] Anand SS, Tarnopolsky MA, Rashid S, Schulze KM, Desai D, Mente A, et al. Adipocyte hypertrophy, fatty liver and metabolic risk factors in South Asians: the Molecular Study of Health and Risk in Ethnic Groups (mol-SHARE). PLoS One. 2011;6:e22112. 10.1371/journal.pone.0022112 PMC3145635 21829446

[16] Sniderman AD, Bhopal R, Prabhakaran D, Sarrafzadegan N, Tchernof A (2007). Why might South Asians be so susceptible to central obesity and its atherogenic consequences? The adipose tissue overflow hypothesis. Int J Epidemiol.

[17] Korenblat KM, Fabbrini E, Mohammed BS, Klein S (2008). Liver, muscle, and adipose tissue insulin action is directly related to intrahepatic triglyceride content in obese subjects. Gastroenterology.

[18] Fabbrini E, Magkos F, Mohammed BS, Pietka T, Abumrad NA, Patterson BW (2009). Intrahepatic fat, not visceral fat, is linked with metabolic complications of obesity. Proc Natl Acad Sci U S A.

[19] Wulan SN, Westerterp KR, Plasqui G. Ethnic differences in body composition and the associated metabolic profile: a comparative study between Asians and Caucasians. Maturitas. 2010;65:315–19. 10.1016/j.maturitas.2009.12.012 20079586

[20] Popkin BM (2001). The nutrition transition and obesity in the developing world. J Nutr.

[21] van Herpen NA, Schrauwen-Hinderling VB, Schaart G, Mensink RP, Schrauwen P. Three weeks on a high-fat diet increases intrahepatic lipid accumulation and decreases metabolic flexibility in healthy overweight men. J Clin Endocrinol Metab. 2011;96:E691–5. 10.1210/jc.2010-2243 21252252

[22] van der Meer RW, Hammer S, Lamb HJ, Frolich M, Diamant M, Rijzewijk LJ (2008). Effects of short-term high-fat, high-energy diet on hepatic and myocardial triglyceride content in healthy men. J Clin Endocrinol Metab.

[23] Westerbacka J, Lammi K, Hakkinen AM, Rissanen A, Salminen I, Aro A (2005). Dietary fat content modifies liver fat in overweight nondiabetic subjects. J Clin Endocrinol Metab.

[24] Schrauwen-Hinderling VB, Kooi ME, Hesselink MK, Moonen-Kornips E, Schaart G, Mustard KJ (2005). Intramyocellular lipid content and molecular adaptations in response to a 1-week high-fat diet. Obes Res.

[25] Westerterp KR, Wouters L, van Marken Lichtenbelt WD (1995). The Maastricht protocol for the measurement of body composition and energy expenditure with labeled water. Obes Res.

[26] Siri WE (1993). Body composition from fluid spaces and density: analysis of methods. 1961. Nutrition.

[27] Wang Y, Rimm EB, Stampfer MJ, Willett WC, Hu FB (2005). Comparison of abdominal adiposity and overall obesity in predicting risk of type 2 diabetes among men. Am J Clin Nutr.

[28] Greenberg JA (2001). Biases in the mortality risk versus body mass index relationship in the NHANES-1 Epidemiologic Follow-Up Study. Int J Obes Relat Metab Disord.

[29] Kuipers H, Keizer HA, Verstappen FT, Costill DL (1985). Influence of a prostaglandin-inhibiting drug on muscle soreness after eccentric work. Int J Sports Med.

[30] Bonomi AG, Plasqui G, Goris AH, Westerterp KR. Estimation of free-living energy expenditure using a novel activity monitor designed to minimize obtrusiveness. Obesity (Silver Spring). 2010;18:1845–51. 10.1038/oby.2010.34 20186133

[31] Joosen AM, Bakker AH, Zorenc AH, Kersten S, Schrauwen P, Westerterp KR (2006). PPARgamma activity in subcutaneous abdominal fat tissue and fat mass gain during short-term overfeeding. Int J Obes (Lond).

[32] Schrauwen P, van Marken Lichtenbelt WD, Saris WH, Westerterp KR (1997). Changes in fat oxidation in response to a high-fat diet. Am J Clin Nutr.

[33] Schwenzer NF, Machann J, Schraml C, Springer F, Ludescher B, Stefan N, et al. Quantitative analysis of adipose tissue in single transverse slices for estimation of volumes of relevant fat tissue compartments: a study in a large cohort of subjects at risk for type 2 diabetes by MRI with comparison to anthropometric data. Invest Radiol. 2010;45:788–94. 10.1097/RLI.0b013e3181f10fe1 20829704

[34] van Werven JR, Hoogduin JM, Nederveen AJ, van Vliet AA, Wajs E, Vandenberk P (2009). Reproducibility of 3.0 Tesla magnetic resonance spectroscopy for measuring hepatic fat content. J Magn Reson Imaging.

[35] Timmers S, Konings E, Bilet L, Houtkooper RH, van de Weijer T, Goossens GH, et al. Calorie restriction-like effects of 30 days of resveratrol supplementation on energy metabolism and metabolic profile in obese humans. Cell Metab. 2011;14:612–22. 10.1016/j.cmet.2011.10.002 PMC3880862 22055504

[36] Vanhamme L, van den Boogaart A, Van Huffel S (1997). Improved method for accurate and efficient quantification of MRS data with use of prior knowledge. J Magn Reson.

[37] Naressi A, Couturier C, Devos JM, Janssen M, Mangeat C, de Beer R (2001). Java-based graphical user interface for the MRUI quantitation package. MAGMA.

[38] Hamilton G, Yokoo T, Bydder M, Cruite I, Schroeder ME, Sirlin CB, et al. In vivo characterization of the liver fat (1) H MR spectrum. NMR Biomed. 2011;24:784–90. 10.1002/nbm.1622 PMC3860876 21834002

[39] Lear SA, Kohli S, Bondy GP, Tchernof A, Sniderman AD (2009). Ethnic variation in fat and lean body mass and the association with insulin resistance. J Clin Endocrinol Metab.

[40] Ducluzeau PH, Manchec-Poilblanc P, Roullier V, Cesbron E, Lebigot J, Bertrais S, et al. Distribution of abdominal adipose tissue as a predictor of hepatic steatosis assessed by MRI. Clin Radiol. 2010;65:695–700. 10.1016/j.crad.2010.03.013 20696296

[41] Chan DC, Watts GF, Ng TW, Hua J, Song S, Barrett PH (2006). Measurement of liver fat by magnetic resonance imaging: Relationships with body fat distribution, insulin sensitivity and plasma lipids in healthy men. Diabetes Obes Metab.

[42] Wulan SN, Westerterp KR, Plasqui G (2014). Metabolic profile before and after short-term overfeeding with a high-fat diet: a comparison between South Asian and White men. Br J Nutr.

[43] Kotronen A, Seppala-Lindroos A, Vehkavaara S, Bergholm R, Frayn KN, Fielding BA (2009). Liver fat and lipid oxidation in humans. Liver Int.

[44] Sparks JD, Sparks CE (1990). Insulin modulation of hepatic synthesis and secretion of apolipoprotein B by rat hepatocytes. J Biol Chem.

[45] Lear SA, Humphries KH, Kohli S, Chockalingam A, Frohlich JJ, Birmingham CL (2007). Visceral adipose tissue accumulation differs according to ethnic background: results of the Multicultural Community Health Assessment Trial (M-CHAT). Am J Clin Nutr.

